# Exploring health behaviors and the role of pet dogs in households with autistic children: the DANE study

**DOI:** 10.3389/fped.2023.1153124

**Published:** 2023-07-14

**Authors:** Janna R. Adkins, Christina M. Mulé, Deborah E. Linder, Aviva Must, Sean B. Cash, Sara C. Folta

**Affiliations:** ^1^ Friedman School of Nutrition Science and Policy, Tufts University, Boston, MA, United States; ^2^ Division of Developmental and Behavioral Pediatrics, Department of Pediatrics, University of Rochester School of Medicine & Dentistry, Rochester, NY, United States; ^3^ Division of Developmental and Behavioral Pediatrics, Department of Pediatrics, Tufts University School of Medicine, Boston, MA, United States; ^4^ Cummings School of Veterinary Medicine, Tufts University, North Grafton, MA, United States; ^5^ Department of Public Health and Community Medicine, Tufts University School of Medicine, Boston, MA, United States

**Keywords:** autism spectrum disorder, animal-assisted intervention (AAI), human-animal interaction (HAI), nutrition, physical activity

## Abstract

**Introduction:**

Autism spectrum disorder (ASD) often presents a unique set of risk factors that impact healthy eating and physical activity. Animal-assisted interventions (AAI) are a promising approach for autistic children. There is growing evidence for the positive impact of AAIs on self-regulation, which is necessary for initiating and maintaining behavioral changes. Pet dogs offer several potential advantages as a vehicle for an AAI focused on health behaviors. However, little is known about the experiences of autistic children and their families with respect to dog ownership and the mechanisms through which such an AAI might operate.

**Methods:**

We conducted interviews with ten parent-child dyads to explore the role of pet dogs in the lives and lifestyle habits of families with an autistic child. Interview guides were designed to explore the relationship between the autistic child and the pet dog and the role of the dog in family life; attitudes and practices related to physical activity and nutrition; and thoughts about intervention strategies. We used a directed qualitative content analysis approach for analysis.

**Results:**

Themes indicate a strong bond between the child and the dog, the child's enjoyment in caring for their dog, and successful integration of dogs within family routines. In contrast, minor themes emerged around the challenges that owning a pet dog posed for families with an autistic child. In terms of nutrition and physical activity, a major theme among children was that healthy eating and exercise were important for both them and their dogs. However, minor themes suggest challenges with healthy eating and exercise and room for improvement for these behaviors. Parents held favorable views toward an intervention that would incorporate the family dog to teach children about nutrition and physical activity, although they expressed some concerns about feasibility.

**Discussion:**

This exploratory work suggests that AAIs to improve nutrition and physical activity could build on the strong bond that children have with their pet dogs, but should consider the specific needs of each family, including the needs of the pet dog.

## 1. Introduction

Increasing evidence suggests that children with autism spectrum disorder [ASD; person- and identity-first language is used intentionally in the remainder of this paper in recognition of the autistic community's right to self-determination (1)] are at greater risk for obesity compared to their neurotypical counterparts (2–5). This disparity may be mediated in part by behavioral and sensory challenges that impact eating and physical activity behaviors. For example, atypical eating patterns, food rituals, and hypersensitivity to tastes, textures, and smells are manifestations of ASD that are likely to contribute to poor diet quality (6). Notably, autistic children tend to choose foods that are higher in calories and lower in nutrients and fail to meet recommendations for fruit and vegetable intake (7). Moreover, physical activity may be avoided because of difficulties with gross motor skills (8), the social interactions that are required for many activities (9), and the intense sensory and visual stimuli that are typical in settings where physical activity occurs (10). Very few obesity prevention interventions exist that are designed to take into consideration the attributes of ASD (11–13), and fewer still offer family-based lifestyle programming. Novel approaches are needed to address this gap.

Animal-assisted interventions (AAI) are a broad category of interventions that use various animal species to benefit humans, and can include therapies, education, or activities (14). There is a growing body of research on the positive impact of animal-assisted interventions (AAI) on self-regulation (15, 16), which is necessary for initiating and maintaining behavioral changes (17), including changes in diet and physical activity (18). In addition to enhancing self-regulation, an AAI may promote physical activity and healthful nutrition behaviors in several ways that could help address the specific barriers faced by autistic children. For example, an AAI could introduce nutrition-related concepts with an emphasis on the animal's health to increase nutrition knowledge and awareness in a less stressful way since the focus is not on the child's eating behaviors; or an animal's non-judgmental attitude and patience could be emphasized with a child with gross-motor skill deficits, encouraging participation in physical activity.

While certified therapy animals are often used for AAIs, they are often inaccessible and costly. Pet dogs are an alternative to certified therapy animals for use in AAIs that may be more practical, and as members of the family environment they remain after an intervention concludes, potentially resulting in greater sustainability (19). There is some correlational evidence from the general population that dog ownership may promote physical activity among children (20).

While AAIs that incorporate pet dogs for obesity prevention in families with an autistic child represents a promising and novel approach, given a lack of literature in this area, formative work is needed to understand the household dynamics and the best approaches to such an intervention (19, 21). The purpose of this qualitative study was to gain insights into children's relationships with their pet dog to understand if it might be leveraged in an AAI focused on health behaviors; how the pet dog integrates into households with an autistic child to determine the appropriateness and feasibility of an AAI that would involve the family dog to help promote healthy nutrition and physical activity among autistic children; and parent reactions to this type of AAI.

## 2. Methods

The multidisciplinary team included a PhD-level researcher with expertise in community-based strategies for improving dietary intake and physical activity as well as expertise in qualitative methods, including adapting methods to meet the needs of autistic people (SCF); a student pursuing a doctorate in nutrition interventions, communications, and behavior change (JA) who was trained in qualitative methods by the qualitative expert; a PhD-level pediatric psychologist who has extensive expertise in the diagnosis and treatment of ASD and physical activity promotion in ASD (CMM); a board-certified veterinary nutritionist with knowledge and experience in AAI and pet obesity (DL); a PhD-level researcher who focuses on observational and intervention studies of obesity in vulnerable populations, with an emphasis on youth with developmental disabilities (AM); and a PhD-level behavioral economist whose work focuses on the economic aspects of behavior around food and nutrition, including how children behave when engaging in autonomous food purchasing activities, who is also the parent of an autistic child (SBC).

We conducted interviews with a purposive sample of ten parent-child dyads to better understand the role of pet dogs in the lives of families with a child who is diagnosed with ASD and the potential to conduct an AAI on health behaviors that involves the pet dog. This sample size helped assure that we would achieve data saturation (22). To be eligible for the study, the dyads were required to live with a pet dog, be English-speaking, and have a child between 8 and 18 years of age with an ASD diagnosis and intelligence quotient score greater than 70. We recruited the dyads through recruitment emails to veterinary school and a therapy dog organization listservs, social media (e.g., Facebook), and targeted recruitment of clinic patients who were known to be autistic.

The research team collaborated with stakeholders to develop semi-structured interview guides (21). The stakeholder panel included: a provider of AAI; two Board Certified Behavior Analysts (BCBAs); two parents of autistic children; and an autistic young adult, along with his mother. The interview guide for children included three topic areas. The first was designed to understand the child's relationship with the pet dog to help assess the appropriateness of an AAI that incorporates the pet dog might and how an AAI might be best designed to build on the existing relationship. In this area, we also asked about responsibilities for taking care of the dog and activities with the dog to understand how lessons about nutrition and physical activity might be incorporated into these activities. The second topic area was about the eating and exercise habits of the dog and the child. This topic area was designed to help shape the informational content that would be most relevant in an AAI. It was also designed to understand children's attitudes toward healthy eating and physical activity for themselves and for their dogs as this would form the basis for motivation to participate in an AAI on these topics. In the third topic area, children were asked directly for their thoughts on learning more about physical activity and nutrition for their dogs. The interview guide for parents covered the same topic areas, and in addition: perceptions about the effect of autism on child-dog interactions, fitting the dog into the family routine, the impact of the dog on the family, and reactions to a potential AAI. The questions about fit with the family routine and impact of the dog on the family were included to help understand the appropriateness and feasibility of an AAI the includes the pet dog. Because of the timing of the study, we also asked parents about the impact of the COVID-19 pandemic on these areas. See Supplementary Tables S1A and S1B for major topics and questions for both guides. Demographic data were collected during screening to help characterize the sample.

The interviews were conducted from August 2020 to December 2020 using the Zoom videoconferencing app. The parent interviews were conducted by a graduate student on the team (JA), and the child interviews were conducted by the qualitative expert (SCF) with experience interviewing autistic youth. The study was approved by the Tufts University Social, Behavioral and Educational Research Institutional Review Board. Parents provided verbal informed consent for themselves and their child prior to the interviews. Child assent was obtained verbally prior to each child interview. Interviews were designed to last no longer than 1 h, and each dyad received a $50 gift card and dog toy upon completion of their interviews.

All interviews were audio-recorded on Zoom and transcribed verbatim. We used a directed qualitative content analysis approach. We developed an initial codebook based on the interview guides and added codes based on review of the transcripts. NVivo 12 (QSR International Pty Ltd., 2018) was used to assist with the analysis. Primary coding was done by JA under the supervision of SCF. These team members established inter-coder reliability based on double-coding of one child transcript and one parent transcript. Satisfactory agreement was established (average Cohen's kappa was >0.9 for both the transcripts). Minor differences were discussed, and the codebook was revised accordingly, mainly by clarifying code definitions. The codebook remained stable at this point, reflecting code saturation (22). Major themes were developed based on similarities in responses in at least six of the ten transcripts, and minor themes were based on similarities in 3–5 of the transcripts. To finalize the themes and interpret results, findings were discussed with the study team and stakeholders.

## 3. Results

### 3.1. Participants

Fifteen parents contacted the study in response to recruitment efforts. Two did not meet eligibility criteria. Of the 13 eligible parents, two did not respond to scheduling follow-ups and one indicated that the study procedures would be difficult for her child and therefore declined to participate. Interviews were scheduled and completed with the remaining ten parent-child dyads. The characteristics of the participants are described in Table 1. Of the child participants, the median age was 9 years; 8 were male, 1 was female, and 1 was other. Of the parent participants, all were female and non-Hispanic white, 8 were married, and 2 were divorced. Three of the parent-child dyads were eligible for free or reduced lunch, and the number of children in the household ranged from 1 to 6. The breed of the dogs varied. Dyad 2 was the only family that had more than one dog. All families had acquired their dog at least one year ago except Dyad 2, which had acquired a second dog six weeks prior to the interviews.

**Table 1:** Characteristics of parent-child dyads.

Dyad #	Child age (years)	Child gender	Parent gender	Parent race/ethnicity	Parent marital status	No of children in household	Free/reduced lunch eligible?	Pet dog breed and sex
1	8	Male	Female	Non-Hispanic White	Married	1	No	Rottweiler, male
2	8	Female	Female	Non-Hispanic White	Married	2	No	Pit bull mix, female
3	9	Male	Female	Non-Hispanic White	Married	2	No	Boxer mix, male
4	17	Male	Female	Non-Hispanic White	Divorced	1	Yes	Lab Chow Coon mix, male
5	8	Male	Female	Non-Hispanic White	Divorced	1	No	Pomeranian, male
6	13	Male	Female	Non-Hispanic White	Married	6	No	Australian Cobberdog, female
7	9	Male	Female	Non-Hispanic White	Married	2	No	Chihuahua Maltese mix, male
8	10	Other	Female	Non-Hispanic White	Married	2	No	Goldendoodle, male
9	16	Male	Female	Non-Hispanic White	Married	3	Yes	Lab mix, female
10	9	Male	Female	Non-Hispanic White	Married	2	Yes	Mini poodle, male

### 3.2. Themes

The directed qualitative content analysis approach is largely deductive, and most themes arose directly from the questions asked. Based on the data, we combined our findings topically into three major domains: (1) child's relationship with the family dog; (2) the role of the dog in families with an autistic child; and (3) eating and exercise habits, including reactions to a potential AAI involving the pet dog. A summary of findings is provided in Table 2. For clarity we have underlined names and pronouns within quotes when they refer to the pet dog.

**Table 2:** Summary of themes.

Domain	Sub-domain	Themes	
Child's relationship with the dog	Positive	Child, major	Spending time with dog makes them happy
		Parent, major	Child has a strong bond with the dog
		Parent, minor	Helps with challenges related to autism
	Negative	Child, major	Some negative aspects to playing with dog (e.g., love biting and licking)
		Parent, minor	Autism negatively impacts interactions with dog (e.g., getting frustrated easily)
Role of the dog in families with an autistic child	Integration with family routine	Parent, major	Typical part of family routine; schedule fits well
		Parent, minor	Some challenges to having a dog (e.g., added responsibilities)
	Caring for dog	Parent, major	Children view caring for dog as part of routine and enjoyable
		Child, major	Taking care of dog is important—member of the family
		Parent, minor	Taking care of dog makes child feel proud
Eating and physical activity	Nutrition and physical activity and the dog	Child, major	Nutrition and physical activity are very important for the dog
	Nutrition and the child	Child, major	Eating healthfully is important
		Parent, major	Healthfulness of child’s diet is good, but room for improvement
		Parent, minor	Pandemic increased food intake and decreased quality of foods eaten
	Physical activity and the child	Child, major	Physical activity is important
		Child, minor	Physical activity is unpleasant
		Parent, minor	Child does not enjoy physical activity
		Parent, minor	Child enjoys physical activity but needs encouragement to participate
	Potential AAI	Child, major	Interested in learning more about nutrition and physical activity for the dog from parents or reading materials
		Parent, major	Favorable perception of this type of program
		Parent, minor	Concerns with specifics of implementation

#### 3.2.1. Domain 1: child's relationship with the family dog

A major theme among children was that playing with and spending time with their dog makes them feel happy. This was often because children perceived their dogs as cute and comforting.

“*He’s really cute. He comes into my bed every morning. When he doesn't find me, he goes around looking around the entire house. He goes and gets my parents. He’s just the cutest. I love him because every time I'm feeling down or something…he comes over and lays on me and licks me. That*’*s the dog he is. We look out for each other.*” *(Child, Dyad 1)*

Like the children, parents also spoke positively about the relationship between their child and the family dog, and the major theme among parents was that there was a strong bond between the child and the dog, due mainly to the protective, attentive, and gentle nature of their dogs. They also said that their children enjoyed the dogs’ calming nature and love.

“*I think the fact that they're both very loving [contributes to the strength of their bond]. Chloe is a very loving and affectionate dog and [child] happens to be a very loving and affectionate kid. She really likes that affection, so she seeks it regularly. I think that*’*s one of the reasons why she loves Chloe so much because Chloe is so interested in her and so affectionate with her, so she gives that mutual affection back if that makes sense.*” *(Parent, Dyad 2)*

“*Toby*’*s gentleness and his tolerance [contribute to the strength of the bond]. He doesn't bark at kids or he doesn't whimper or growl, or anything like that when you're petting him. He’s very, very calm.*” *(Parent, Dyad 3)*

“*They play together. [Child] lays on the bed with him and while he*’*s listening to music on his phone, he lays by the side of Bruce. …Very close bond.*” *(Parent, Dyad 4)*

A minor theme among parents was that the dog’s attention, companionship, and bonding time helped their children with the challenges resulting from autism.

“*[My child] is more verbal now. We've only had Oreo since May, so he*’*s had the verbal skills pretty steadily. In the past, the verbal was really, really difficult for him.*” *(Parent, Dyad 5)*

“*I think [autism*’*s effect on their relationship] is just positive…I think he feels really judged by others, and she doesn't judge him. She loves everybody and she wants to be with everybody, and she loves anybody*’*s attention. There*’*s always positive feedback that she gives him that he doesn't get from everybody else necessarily.*” *(Parent, Dyad 6)*

Although the major themes indicate a very positive relationship between the child and the dog, most children also described some negative aspects to playing with their dog. These varied and included leash tugging, love biting and licking, playing rough with toys, and taking up free time. A minor theme among parents was that autism negatively impacts their children's interactions with the dog. Problematic behaviors included picking at the dog's face, walking loudly, or getting frustrated easily.

“*I think that the autism and some of the behaviors, like just being a little bit louder, more physically, where he*’*s not really aware of his physical body, sometimes he comes into a room and the arms are doing this [physical gesture], and it just scares the dog. I feel like I'm constantly managing the relationship between the two of them.*” *(Parent, Dyad 7)*

“*I think the only time if there*’*s any problematic things is sometimes she'll get frustrated if Chloe won't come over to her. She has really low frustration tolerance.*” *(Parent, Dyad 2)*

Since this study took place during the COVID-19 pandemic, parents also gave insight on how the relationship between their child and family dog had changed due to the pandemic. Many of them mentioned that their child spent more time with the dog, and they were trying to spend more time outside. According to the parents, most children enjoyed the extra time and reported that their child spends a range of 10 min to 4 h per day with the family dog.

“*[Their relationship] is probably just stronger. They spend more time together and there are fewer distractions or opportunities for us to do something that Enzo wouldn't be included in. We haven't been to a museum or a shopping trip for an out-of-town weekend trip in almost a year. They are just spending more time together and getting that much closer.*” *(Parent, Dyad 1)*

#### 3.2.2. Domain 2: role of the dog in families with an autistic child

A major theme among parents was that the pet dog is a typical part of the family's routine, and their dog's schedule fits well with the family's. Parents stated that they take the dog with them wherever they can, which includes outdoor activities such as walks around the neighborhood, hikes, and going to the park. Parents described facilitators to fitting the dog into the routine, which included the dog's temperament and size, aligning the dog's schedule with the family's schedule, and placing a high importance on including the dog in family activities.

“*[The dog fits into our routine] really nicely actually. [chuckles]…You get up, you go to the bathroom, you take the dog out. You eat breakfast and feed the dog. It added an additional step to learn, but it fits into the flow. Right after lunch, take the dog out. Right after school, take the dog out. It was a step that we added but it fit in pretty regularly.*” *(Parent, Dyad 8)*

“*There are certain activities that we can't include him in, like swimming in a pool but we live two or three blocks from [lake] so there were plenty of days over the summer that we would walk down to the beach and take him off his leash and he swam with us.*” *(Parent, Dyad 1)*

“*She walks very nicely on a leash, she’s a very good listener. She'll literally walk right beside you on the leash.*” *(Parent, Dyad 2)*

*“He’s easy to take with, he’s very small. As far as when we go walking, it*’*s mainly on the property here, we don't go too far out.*” *(Parent, Dyad 5)*

Although the ready integration of the dog within the family's routine was a major theme, there was also a minor theme related to challenges. These included added responsibilities, financial concerns, and managing the dog and child's relationship.

“*Obviously, financially, that*’*s a concern… she just had heart surgery, so that was quite an expense. That was fine. Where we are, it was okay, but it was still an added expense. I can't think of anything else.*” *(Parent, Dyad 6)*

“*[Having a dog] is a lot of extra life skills as a parent. I don't think I anticipated how much longer it would take [the child] to understand it. That*’*s really the only challenge, is the repetition to get [the child] to understand the process, but that*’*s partly just [child]*’*s limitations. He*’*s not a multiple-step kiddo. Feeding a dog is sometimes multiple steps, which is hard.*” *(Parent, Dyad 8)*

In terms of children caring for the dog, a major theme among parents was that their children view this as part of a routine and enjoyable. Among the children, a major theme was that taking care of their dog is important because the dog is a part of the family whom they care about. Parents said they help their child, or their child is expected to help them, with feeding, watering, walking, and/or brushing the dog. From the children's perspective, their main responsibility was playing with the dog. Of the ten parent-child dyads, only one child stated that they do not take care of the family dog at all.

“*[I take care of my dog by] giving her some food, playing outside with her sometimes.*” *(Child, Dyad 6)*

“*They're responsible for feeding, brushing, walking. When we do a bath, they're responsible for at least helping towel-off afterwards. It*’*s hard for a 10, almost 11-year-old, to do those responsibilities, but it*’*s part of ownership.*” *(Parent, Dyad 8)*

A minor theme among parents was that the dog care responsibilities made their child feel proud and better about themselves.

“*I think he enjoys [his responsibilities], especially now being home gives him something to do and being responsible. I think it helps him feel better about himself.*” *(Parent, Dyad 4)*

“*I think [feeding and watering the dog] makes him feel good, but it*’*s also just very normal at this point. Like the same way that he is expected to get himself breakfast, he knows that Enzo needs to eat too. There*’*s a little bit of pride involved, but it*’*s also just part of his routine at this point, which is a big thing for him.*” *(Parent, Dyad 1)*

#### 3.2.3. Domain 3: eating and physical activity for the child and their dog

A major theme among children was that good nutrition and physical activity are very important for the family dog.

“*Yes, [it*’*s important for Bailey to eat healthy foods]. A dog*’*s health is one of the most important things when it comes to pet care.*” *(Child, Dyad 9)*

“*[It*’*s important for my dog to exercise] because he needs to have big, strong dog bones to be healthy so he could become an even better boy and take over the world.*” *(Child, Dyad 7)*

“*I think it*’*s important for him to get exercise. Like I said, he needs it to develop his growth. I can tell you he also really enjoys it. It helps build up him wanting to be outside more and more.*” *(Child, Dyad 1)*

Children also felt that healthy eating is important for themselves because it keeps their body healthy.

“*When it comes to my condition, I see [healthy eating] as a very important thing because nutrition is one of the most essential things when it comes to growth of the body and enhancement of your internal systems.*” *(Child, Dyad 9)*

“*Yes, [it*’*s important to eat foods that are good for me]. It helps my body stay healthy*” *(Child, Dyad 5)*

However, children generally did not enjoy eating healthy foods as much as they felt it was important. Among parents, a major theme was that the healthfulness of their child's diet was good but could be better. Introducing new foods and textures and getting their child to choose healthy foods on their own was a common challenge. When asked about how the pandemic may have impacted eating habits, a minor theme was that the children's food intake increased due to a lack of structured school mealtimes and being at home.

Similar to the theme around eating healthfully, a major theme related to physical activity for children was that it is important because it keeps them healthy and helps them grow.

“*For me, [exercise] is really important because if a human doesn't get exercised, then, as I said, their muscles could get really weak.*” *(Child, Dyad 10)*

Children and parents described a range of physical activities that children engaged in, which commonly included bike riding and playing outside. When asked about the impact of the pandemic, families were divided between those reporting that their child got less physical activity due to the lack of structured activity at school and being inside more often, and those saying their child increased their physical activity because of being at home more and making an extra effort to get moving. A minor theme was that physical activity is unpleasant: some children said it makes them tired, they would rather be doing other things, they are not good at it, or they simply do not like it. Parent perceptions were generally consistent with their children's. A minor theme among parents was that their children enjoy exercise but need some encouragement to participate.

“*If something naturally incorporates exercise, he loves it. If you make something to him look like and sound like exercise, he doesn't want to do it, but if you have the same exact activity but call it a relay race or call it an obstacle course, he*’*s all about it.*” *(Parent, Dyad 10)*

Parents and children were asked to provide their thoughts about programming that would incorporate the family dog to help teach children about nutrition and physical activity. Children were asked whether they would be interested in learning more about nutrition and physical activity for their dogs, since the potential program would be designed to introduce nutrition and physical activity-related concepts through the lens of the dog's health. A major theme was that children were interested in learning more, either from their parents or by practicing or reading about it. For the few children who were not interested, reasons were that the children are not primary caretakers of the dog, or the child felt that their dog is in a good place regarding healthy eating and exercise.

“*I think maybe [learning more about how to feed Bailey healthy foods] is something important. That actually sounds interesting. I'll go with that. I care about her as much as anyone else.*” *(Child, Dyad 9)*

“*Yes, [I would like to learn more about how to feed Cooper healthy foods]. I would like to learn what dogs eat and how much food you should give a little dog. (Child, Dyad 10)*

“*[I’m] not exactly [interested in learning more about how to feed my dog healthy foods] because I don't exactly care for him that much.*” *(Child, Dyad 7)*

It was similarly a major theme among parents that they liked the idea of this type of program. Many couldn't think of any potential drawbacks, and some felt that it would also teach their child about responsibility. The parents whose child had an Applied Behavior Analysis (ABA) therapist liked the idea of the therapist implementing the program.

“*Right now, [doing the program] would be hard because it*’*s over Zoom and she*’*s [ABA therapist] not in person but the benefits would be great. They would really teach him responsibility and self-confidence and self-esteem.*” *(Parent, Dyad 3)*

“*[The benefits of the ABA therapist doing the program is], it*’*s not mom and dad. It*’*s not the primary caregiver. It*’*s not someone else asking them almost like a chore. It*’*s a,* “*Hey, let*’*s try.*” *It becomes a game and fun and something a little more treat-based or exciting-based for them because it wouldn't be mom or dad or aunt or uncle, grandma, grandpa, whoever they're living with.*” *(Parent, Dyad 8)*

Although reaction to the program was largely positive, parents expressed some concerns about implementation. For parents with an ABA therapist, there was some concern about the relationship between the therapist and the dog: the therapist would need to feel comfortable and would also need to understand how to best care for the dogs’ needs as well as the children's. Some parents without an ABA therapist perceived the potential for the program to be overwhelming if they were the ones implementing it. Parents offered advice on program development, such as making sure that parents had information on the goals of the program as well as finding a way to make the program specific to each child but also generalizable.

“*[Information I want as a parent] is specific ideas on exactly what we should be doing. Tell me specifically what you want me to do. I just think it*’*s so hard sometimes to come up with– If you're trying to incorporate nutrition and exercise with kids and how that works with the dog, just tell me what should I be doing. You want me to go outside with them? Do you want me to do this activity? Maybe some ideas. Give me some activities that you want me to try, and how do I incorporate the nutrition aspect with the dog? That*’*s a little bit confusing to me.*” *(Parent, Dyad 7)*

“*[I want] those step-by-step instructions so I can learn more information about it.*” *(Parent, Dyad 4)*

“*The more information that you guys could give to the family about how this can benefit the family as a whole, I think would probably be good information to have.*” *(Parent, Dyad 10)*

## 4. Discussion

Findings from our qualitative study suggests a positive role that pet dogs have for families with an autistic child. Children enjoy the bond with their dog and the dog's affection and attention while parents value how the dog integrates well into family life. Leveraging this relationship and the unique role of pet dogs in the family may be a promising approach to improving nutrition and physical activity among children with autism, and findings suggest several ways an AAI might be implemented.

Our findings suggest that an AAI that incorporates pet dogs to teach children about nutrition and physical activity is likely to be feasible and appropriate, at least in some families. While a pet dog could potentially introduce additional responsibilities and burden to families with an autistic child, a major theme was that pet dogs are a typical part of the family's routine and their dog's schedule fits well with the family's. Parents said that it was relatively easy to align the family schedule with the dog's, and that they often prioritized activities that could include the dog. Similarly, several studies have found that pet dogs have a positive impact on parent stress and family functioning in families with an autistic child (23–25), lending support to the appropriateness of an AAI. However, the minor theme related to challenges of having a dog are consistent with Carlisle et al. (26), in which they identified time and cost of care as challenges related to dog ownership. In implementing an AAI, it may be important to consider the strain that the dog may already be adding to some families.

Our findings related to the child-dog relationship is consistent with other literature and further suggests the appropriateness of an AAI that incorporates the pet dog. Studies with typically developing children have similarly found that children have a strong bond with the family pet (27). While there is scant literature on the relationship between autistic children and their pets, our findings are consistent with four studies in which parents also reported children's strong bonds with their pets, including dogs (26, 28–30). In one study, however, children who had lived with a pet (dogs, cats, or rodents) since birth demonstrated less bonding than children who acquired one later (31). In terms of an AAI, the children's concern for the dog's health could provide a strong motivator for participation in such a program. The strong bond with pet dogs could also offer an avenue to indirectly model healthy eating and physical activity behaviors for their child companions. However, this approach may not be feasible for children who feel less connected to or responsible for their dog's health and well-being. Some parents in our study also identified a need to manage the relationship between the dog and the child as a challenge; problematic behaviors such as meltdowns and invasion of the dog's personal space have been noted in other studies as well (29, 32). In considering an AAI involving the family dog, it will be critical to consider the relationship between the child and the dog, including any history of negative interactions, to ensure the safety of both. It is also important to consider the temperament and needs of the pet dog to help ensure that an AAI is a good fit and safe for both the child and the dog. As in our study, other studies indicate that it is common for autistic children to be responsible for some aspects of the care of their pets (26, 30, 31). Although not conclusive, some evidence suggests that taking responsibility for pets could increase social functioning (31) and decrease depressive symptoms (30). In terms of developing an AAI, children's involvement with their dog's care, including feeding and walking, could provide an opportunity for lessons about nutrition and physical activity for both dogs and humans.

Most parents liked the idea of a dog-assisted nutrition and physical activity intervention for their child, but some were concerned about time constraints and possibly feeling overwhelmed if they were to implement it themselves. In contrast, parents liked the idea of an ABA therapist delivering the program. A program delivered by ABA therapists could be beneficial if a standardized program is integrated into therapists’ typical workflows. This integration could allow for nationwide dissemination and impact among families with children with autism. However, factors to consider are the uniqueness of each family and the skill and comfort level of the ABA therapist, who would need to address safety for both the child and the dog.

This study helps address a lack of literature on the role of the pet dog in a family with an autistic child. It also provides information to help assess the appropriateness of an AAI on nutrition and physical activity that incorporates pet dogs and to help shape what such an AAI might entail. The study team included a comprehensive range of expertise relevant to the study question. It also included members with lived experience, both on the study team and in the stakeholder panel that helped inform the work. Another strength of the study was the inclusion of both children and parents. Most qualitative studies have relied solely on parents to provide their own and their children's perspectives (33). The sample size is deliberately small in this qualitative study as we sought to gain in-depth insights. We also achieved code saturation (22), suggesting the robustness of themes among the voices included in our sample. The sample included perspectives from families in several U.S. states, as well as a range of voices in terms of child age and family structure and socio-economic status. The gender identities of the children are reflective of the ASD population (34, 35). However, the sample was limited to the inclusion of mothers only. Fathers of autistic children have been underrepresented in the literature and their perspectives may be different. The sample was homogeneous in terms of race/ethnicity and therefore not reflective of the general population of families with autistic children in this way (34). Future studies are needed to gain perspectives from participants that more fully represent the ASD population. The study sample was limited to children with the verbal skills to participate in the interview and who did not have an intellectual disability. The inclusion of children with more significant verbal communication challenges would have required adaptations to the qualitative methodology that was beyond the resources of this study. However, it will be important to work toward adapting the methodology so that a greater range of stakeholders may be included in future work. In this study, we also chose to limit the sample to children for whom the type of AAI being proposed would be most appropriate. Future work may consider how to develop an AAI to promote healthy eating and physical activity for a broader range of autistic children, although it may be appropriate to involve other types of animals or trained therapy dogs rather than pet dogs. Another limitation of this study is that by nature of the interview topic, our sample may be biased toward families that were more likely to have had positive experiences with the family dog in the context of nurturing the growth and development of an autistic child. It is important to note that a nutrition and physical activity AAI may be appropriate and feasible when a pet dog is already well-integrated into the family and the child has bonded well with the dog. It would not necessarily be advisable to incorporate a new dog into families for the purpose of such an AAI, especially if the autistic child exhibited aggressive behaviors toward or a dislike of animals, or if a pet dog would pose a greater burden to the family.

This study contributes an understanding of the experiences of autistic children and their families with respect to dog ownership and the potential mechanisms through which an AAI on nutrition and physical activity that incorporates pet dogs might work. Findings lend support to the potential of incorporating the family dog into an AAI, an approach that is potentially more accessible and sustainable than AAIs that use therapy animals. Future studies may build on this foundation to develop and test AAIs for obesity prevention among autistic children.

## Data Availability

The datasets presented in this article are not readily available because raw data may not be shared outside of the research team per IRB. Requests to access the datasets should be directed to sara.folta@tufts.edu.
