# Supplementary material for: Exploring health behaviors and the role of pet dogs in households with autistic children: the DANE study
Source: Front Pediatr. 2023 Jul 14;11:1153124. doi: 10.3389/fped.2023.1153124 (PMC10376699; doi:10.3389/fped.2023.1153124)
Supplement: Supplementary file 1 [file Datasheet1.pdf]

Supplementary Material

Exploring Health Behaviors and the Role of Pet Dogs in Households with Autistic Children: The DANE Study

Janna R. Adkins, Christina M. Mulé, Deborah E. Linder, Aviva Must, Sean B. Cash, Sara C. Folta*

*** Correspondence:** Sara C. Folta, PhD: email@uni.edu

Supplementary Table 1a. Semi-Structured Interview Questions for Children

First, can you tell me about your dog? [Probes: What kind is it? What's his/her name? How long have you had him/her?]

Questions about child's current relationship with the pet dog

1. How much time do you spend time with your dog every day? [Probe: This is time that you spend walking, feeding, or playing with your dog.]
    a. Does your family spend part of this time with you and your dog?
    b. Do you spend one-on-one time with your dog?
    c. How do you feel about the time you spend with your dog?
    d. You told me you spend [amount of time] with your dog each day. Would you like to spend more than [amount of time] with your dog? Would you like to spend less than [amount of time] with your dog?
2. Do you take care of your dog? [Probes: By feeding your dog, making sure your dog has water, or taking your dog for a walk]
    a. What do you usually do to take care of your dog?
        i. Who usually takes your dog for a walk?
        ii. Who usually feeds your dog?
    b. Is taking care of your dog important to you?
3. What kinds of things do you like to do with your dog?
    a. Can you tell me more about [activity]?
        i. Do you do that every day?
        ii. How much do you like it?
4. How do you feel about your dog overall?
    a. What do you like about your dog?
    b. Is there anything that you would change about your dog?
    c. Is there anything you don't like about playing with your dog?
5. How can you tell when your dog is upset or stressed?
    a. What do you do when he/she is upset or stressed?

Before we move on to the next set of questions, would you like to take a 5-minute break?

Thoughts on integrating physical activity and nutrition within family life in households that include a pet dog

1. What are some of your dog's favorite foods to eat?
    a. Do you think they are good for your dog?
    b. What makes them good for your dog or not?
2. How about you – what are some of your favorite foods to eat?
    a. Do you think those are good for you or not?
    b. What makes them good for you (or not)?
3. Do you think it's important for your dog to eat healthy foods?
    a. Why do you think it's important/not important?
    b. Does your dog like to eat foods that are good for him/her?
4. How important is it for you to eat foods that are good for you?
    a. Why do you think it's important/not important?
    b. Do you like to eat foods that are good for you?
    c. Is there anything you don't like about eating foods that are good for you?
5. Are there foods that are good for both you and your dog?
    a. What are examples of those?
6. Do you think your dog likes to exercise?
    a. How important is it for your dog to exercise?
    b. Why do you think it's important/not important?
7. For you, how important is exercising?
    a. Why do you think it's important/not important?
    b. How often do you exercise?
    c. What kinds of exercises do you do?
        i. Can you tell me more about [specific exercises]?
    d. Is there anything you don't like about exercising?
8. Do you have a therapist who works with you?
    a. [If yes] What would you think about doing some exercise with your dog, like going for a
    - walk, during your therapist appointments?
        i. What would you like about getting exercise with your dog during your therapist
        - appointments?
        ii. What wouldn't you like about it?
9. Do you want to learn more about how to feed your dog healthy foods?
    a. [If has therapist] What do you think about learning more about this during your therapy appointments?
        i. What would be good about learning more about feeding your dog healthy foods during your therapy appointments?
        ii. What would be bad about learning more about feeding your dog healthy foods during your therapy appointments?
        iii. Are there other ways you would want to learn about feeding your dog healthy foods? [Probes: With parents, by reading about it]
    b. [If doesn't have therapist] How would you want to learn about this? [Probes: Would you want to learn more about feeding your dog healthy foods from your parents? Would you want to learn more about this by reading something about it?]

i. Would you want to learn more about the exercise that your dog needs?

1. How would you want to learn about this? [Probes: Would you want to learn more about the exercise your dog needs from your parents? Would you want to learn more about this by reading something about it?]

Supplementary Table 1b. Semi-Structured Interview Questions for Parents

First, can you tell me about your family's dog? [Probes: What kind is it? What's his/her name? How long have you had him/her?]

Questions about child's current relationship with the pet dog and the dog's place in family life

1. Describe the relationship between your child and your family's dog.
    a. What activities do they do together?
    b. How much time do they spend together?
    c. How much do you think your child cares about the dog?
    d. What does your child enjoy about spending time with the dog?
    e. What does your child not enjoy, if anything?
2. Tell me about your child's responsibilities for taking care of the dog.
    a. What are your child's responsibilities in terms of walking the dog?
        i. [If responsibilities] How does your child feel about these responsibilities?
        ii. [If no responsibilities] In what ways, if any, have you tried to have your child take responsibility for walking the dog?
    b. What are your child's responsibilities in terms of feeding the dog?
        i. [If responsibilities] How does your child feel about these responsibilities?
        ii. [If no responsibilities] In what ways, if any, have you tried to have your child take responsibility for feeding the dog?
3. In what ways, if any, do you think your child's autism affects his/her relationship with your dog?
    a. Describe the bond between your child and your dog.
        i. [If stronger bond] What do you think contributes to the strength of this bond?
        ii. [If not bonded] What, specifically, do you think gets in the way of them bonding?
    b. In what ways, if any, do you think autism causes your child to act in a problematic way toward the dog?
    c. In what ways, if any, do you think autism affects your child's ability to communicate with your dog?
        i. In what ways, if any, does it affect your child's ability to read your dog's stress signals?
    d. What does your child do when the dog is stressed, if anything?
4. For many families with an autistic child, there is a need for a high degree of structure and routine. Describe how your dog fits in with your family's routines.
    a. Describe any challenges with fitting your dog's needs into your family's routine.
5. How much does your family enjoy activities with the dog?
    a. How important is it to your family that the dog is included in family activities?
    b. How involved with these activities is your child with autism?
6. How has having a dog impacted your family overall?
    a. What are positive impacts?
    b. What are challenges and added concerns or responsibilities?

7. How has your child's relationship with the dog changed because of COVID-19, if at all?

Questions about child and family's attitudes, values, and norms related to physical activity and nutrition

1. Tell me about your child's eating habits.
    a. What are his/her favorite foods?
    b. How healthy would you consider your child's diet?
        i. How important is it to you that your child has a healthy diet?
        ii. What are the challenges with having him/her eat a healthy diet?
        iii. What do you do, if anything, to encourage your child to eat healthy?
        iv. How does your child react to your encouragement?
2. In what ways, if any, have your child's eating habits changed during COVID-19?
3. Tell me a little bit about your dog's eating habits.
    a. Is your dog motivated to do things by food?
4. For your family overall, how much of a priority is healthy eating?
    a. What are the challenges to healthy eating?
    b. Is there anything that makes healthy eating easier?
5. Tell me about your child's attitude toward exercise.
    a. How often does he/she exercise?
    b. Describe the activities that he/she enjoys the most, if any.
    c. What does your child not like about exercising, if anything?
6. In what ways, if any, have your child's exercise habits changed because of COVID-19, if at all?
7. Tell me about exercise activities that your family does together, if any.
    a. How often do you engage in these activities?
    b. How important to you is exercising?
    c. What do you not like about exercising, if anything?
    d. Is your dog included in these activities?
        i. What makes it easy to include the dog, if anything?
        ii. Tell me about the challenges to include the dog in exercise activities.

Thoughts on integrating physical activity and nutrition within family life in households that include a pet dog

1. Does your child have an ABA therapist?
2. [*If "yes" to ABA therapist*] Our plan is to develop materials for ABA therapists so that they would be able to incorporate the family dog to help teach children about nutrition and physical activity. For example, the ABA therapist could work with a child on taking the dog for a walk, or they could work on preparing healthy snacks for the dog.
    a. What are your thoughts about this idea?
        i. What do you see as the benefits of having the ABA therapist do this?
        ii. What might be drawbacks?
        iii. What are your concerns?
        iv. What might you want to see as part of this type of program?
    b. Talk about whether you would want your child's ABA therapist to do this with your child.
    c. What information would you want, as a parent, to be able to support this work?
    d. What advice do you have for us as we develop this?

e. Other than working with the ABA therapist, what else might someone developing a nutrition and physical activity program do that would include the family dog?

3. [*If "no" to ABA therapist*] Our plan is to develop a program for that would help families incorporate the family dog into helping to teach children about nutrition and physical activity.
    a. What are your thoughts about this idea?
        i. What would be the best way to deliver a program like this?
        ii. What might you want to see as part of this type of program?
        iii. What might be the drawbacks?
        iv. What are your concerns?
    b. What information would you want, as a parent, to support this work?
    c. What advice do you have for us as we develop this type of program?

1
